# Feasibility of kilohertz frequency alternating current neuromodulation of carotid sinus nerve activity in the pig

**DOI:** 10.1038/s41598-019-53566-8

**Published:** 2019-12-02

**Authors:** Cathrine T. Fjordbakk, Jason A. Miranda, David Sokal, Matteo Donegà, Jaime Viscasillas, Thaleia-Rengina Stathopoulou, Daniel J. Chew, Justin D. Perkins

**Affiliations:** 1 The Royal Veterinary College, Hawkshead Lane, North Mymms, Hatfield, Herts AL9 7TA UK; 2 Galvani Bioelectronics, Translational Sciences, Stevenage, Herts SG1 2NY UK; 3 Present Address: Cathrine T. Fjordbakk, Norwegian University of Life Sciences, Faculty of Veterinary Medicine, PO box 369, Sentrum, 0102 Oslo Norway

**Keywords:** Translational research, Neurology

## Abstract

Recent research supports that over-activation of the carotid body plays a key role in metabolic diseases like type 2 diabetes. Supressing carotid body signalling through carotid sinus nerve (CSN) modulation may offer a therapeutic approach for treating such diseases. Here we anatomically and histologically characterised the CSN in the farm pig as a recommended path to translational medicine. We developed an acute *in vivo* porcine model to assess the application of kilohertz frequency alternating current (KHFAC) to the CSN of evoked chemo-afferent CSN responses. Our results demonstrate the feasibility of this approach in an acute setting, as KHFAC modulation was able to successfully, yet variably, block evoked chemo-afferent responses. The observed variability in blocking response is believed to reflect the complex and diverse anatomy of the porcine CSN, which closely resembles human anatomy, as well as the need for optimisation of electrodes and parameters for a human-sized nerve. Overall, these results demonstrate the feasibility of neuromodulation of the CSN in an anesthetised large animal model, and represent the first steps in driving KHFAC modulation towards clinical translation. Chronic recovery disease models will be required to assess safety and efficacy of this potential therapeutic modality for application in diabetes treatment.

## Introduction

The carotid bodies (CB) are peripheral chemoreceptors responding to changes in arterial blood gases and pH. Chemo-afferent signals travel through the carotid sinus nerve (CSN) to the solitary tract nucleus, inducing respiratory reflexes aimed at restoring blood gas homeostasis^1^. In addition to this well-known respiratory function, recent research has demonstrated that the CB is also a key organ in glucose homeostasis^2^, leading to newfound interest in CB function in relation to metabolic diseases.

In a pre-diabetic rat model, over-activation of the CB was correlated with reduced insulin sensitivity and increased outflow in the sympathetic nervous system, both of which were prevented or reversed by CSN resection^2^. While CSN resection may result in adverse effects, notably the permanent loss of peripheral hypoxic response and decreased CO_2_ sensitivity, other strategies for suppressing CSN signalling without causing permanent neural damage have been investigated^3^. Nerve conduction may be temporarily blocked by applying kilohertz frequency alternating current (KHFAC) electrical stimulation, as action potentials are arrested when they reach the depolarising charge field of the electrode^4^. This mode of CSN conduction-block restored insulin sensitivity and glucose tolerance in a rat model of type 2 diabetes^3^. Both of these metabolic control mechanisms returned to baseline diseased levels within 5 weeks after KHFAC treatment was discontinued, demonstrating a temporary and reversible treatment effect^3^.

While promising as a therapeutic modality for treating metabolic diseases in humans, bioelectronic neuromodulation model development using rodent species have obvious anatomical and engineering limitations. Whereas the rat CSN consists of a single bundle^5^, the human CSN is a complex structure of great anatomical variability and with unpredictable interconnections to other nerves such as the vagus and sympathetic trunk^6^. Also, the ability to achieve conduction-block via KHFAC modulation is based on the need for uniform field distribution across all axons in the target structure, which is harder to accomplish in larger nerves with disparate and dispersed fascicles, due to differences in tissue conductance of surrounding non-neural tissue. Therefore, proof of principle of CSN conduction-block in the rat, is far from proof of principle in humans. Consequently, evaluating the feasibility of CSN neuromodulation in a translational model of relevant size and of similar anatomical complexity, is required for optimal development of the bioelectronic medicine.

Here we demonstrate that the pig is such a model, due to the anatomical similarity between the human and the porcine CSN. The dimensions of the porcine CSN allow implantation of cuff electrodes of human-relevant size, as well as optimising stimulation and blocking parameters for future clinical use. Using an acute anesthetised porcine model, we developed a paradigm of pharmacological- and electrical- activation of the CSN to assess blocking of such activation by applying KHFAC modulation. Our results demonstrate that pharmacological and electrical activation of a chemo-afferent response is reproducible in a human-sized CSN, and more importantly, that blocking such activation through KHFAC modulation is feasible in anesthetised pigs. Observed individual variability in blocking efficiency possibly reflects the anatomical diversity of the CSN, as well as disparate tissue conductances and non-uniform field distribution. These observations may have important clinical implications and should warrant further model refinement of electrodes and paradigms prior to clinical use.

## Results

### Anatomy – the porcine and human CSN are of similar dimensions and anatomical complexity

**Figure 1:**
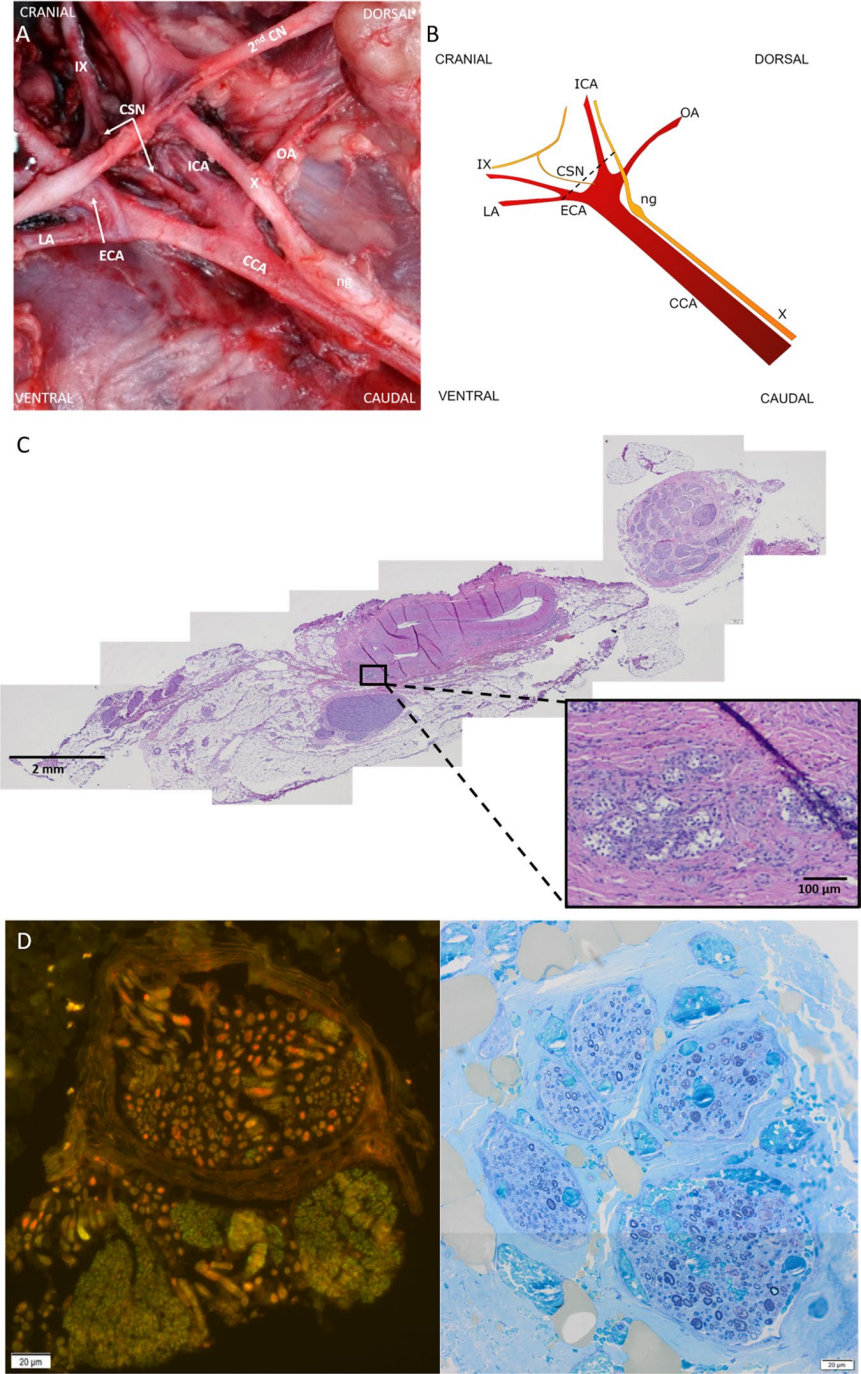
The porcine CSN. (**A**,**B**) Photograph of the carotid sinus region in a cadaver specimen, with a corresponding schematic illustration. Cranial is to the top left, caudal to the bottom right. CCA: common carotid artery. CSN: carotid sinus nerve. ECA: external carotid artery. ICA: internal carotid artery. LA: lingual artery. ng: nodose ganglion. OA: occipital artery. X: vagus nerve. IX: glossopharyngeal nerve. 2^nd^ CN: second cervical nerve. (**C**) Photomicrograph (x2 magnification) taken from a section corresponding to the stippled line in B. At x10 magnification (insert square), CB cells are seen, located in the arterial wall. (**D**) Photomicrographs (x100 magnification) of immunohistochemistry (left) and a semi-thin section (right) of the porcine CSN demonstrating co-localization of ChAT + axons (red) and TH + axons (green) within some fascicles, whereas in other fascicles these two populations are separated. Semi-thin sectioning demonstrates both myelinated and unmyelinated axons, the latter outnumbering the former by a ratio of approximately 5:1.

Prior to conducting physiological experiments, anatomical assessment of the suitability of the pig as a translational model for CSN research was performed. The pig was chosen due to the anatomy of the carotid arterial bifurcation, which closely resembles humans (Fig. 1A,B). As in humans, the porcine CSN was identified as one discrete nerve originating from the glossopharyngeal nerve and terminating in a network of small branches innervating the carotid sinus and the CB. In 4/6 animals, the CSN branched into 2–4 larger branches prior to reaching the carotid bifurcation, where further branching into the above-mentioned network made it difficult to determine whether the larger branches exclusively innervated the carotid sinus or the CB. Total length (11–28 mm), diameter (0.2–2 mm) and branching pattern varied between animals and between left and right sides within animals. Interconnections to the vagus nerve and sympathetic trunk were commonly seen. Dimensions and anatomical complexity were thus comparable to the human where the surgically accessible portion of the CSN is reported in the range of 15–50 mm in length, and 0.5–1.5 mm in diameter^6^.

Histologically, the larger CSN branches consisted of several fascicles (4–9) carrying predominantly small unmyelinated axons (mean minimum Feret’s diameter 1.13 ± 0.4 µm) representing 74.7–89.3% of the total axon number, which corresponds to the proportions reported from other species such as the rat^5^, rabbit^7^ and cat^8^. Mean minimum Feret’s diameter of the myelinated axons were 2.22 ± 1.4 µm, which resembles the human CSN where most of the myelinated axons are within the 2–4 µm range^9^. CSN fascicles were found to be biochemically heterogeneous, as co-localisation of ChAT + and TH + axons were seen in some fascicles, whereas a spatial separation of these were seen in other fascicles. There were small numbers of CGRP + axons irregularly distributed throughout all investigated fascicles, with small amounts of clustering.

### Pharmacological CB/CSN activation evokes consistent respiratory responses *in vivo*

**Figure 2:**
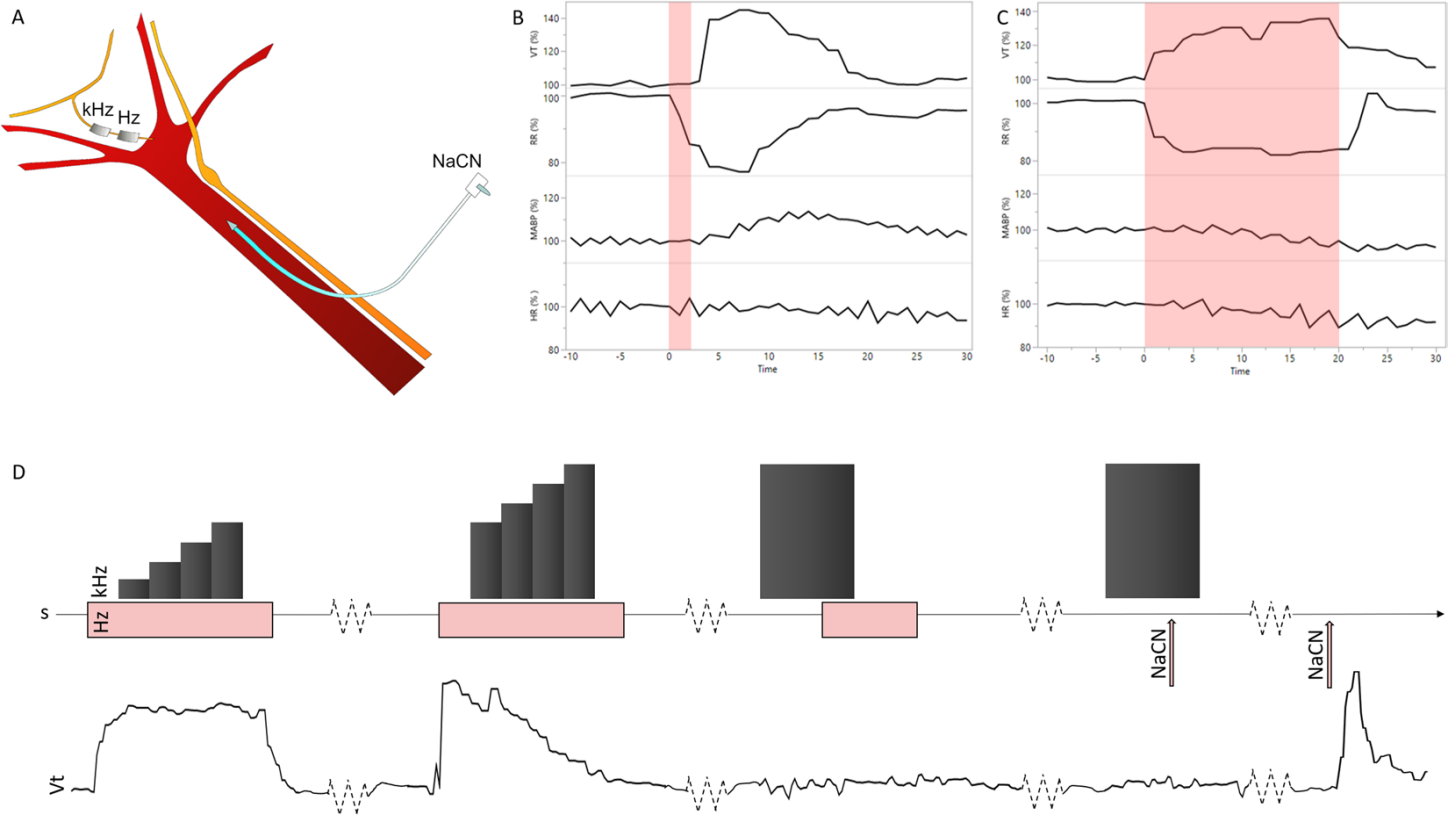
Experimental set-up and model development. (**A**) Schematic illustration of the experimental set-up; an intra-carotid catheter was placed for injection of NaCN, evoking chemo-afferent responses. The CSN was instrumented with two cuff electrodes; the distal (towards the carotid sinus) was used for electrical stimulation at 20 Hz, whereas the proximal (towards the brain) was used for KHFAC blocking at 20 kHz of the electrically or pharmacologically evoked response. (**B**) Representative cardiorespiratory responses evoked by intra-carotid injection of NaCN; the two top panels represent V_t_ and RR, whereas the two bottom panels represent mABP and HR, respectively; responses are calculated as % change from baseline levels. NaCN is injected at time 0; duration of injection is <2 s (pink area). (**C**) Representative cardiorespiratory responses to electrical stimulation of the CSN at the identified respiratory response threshold. Stimulation is delivered for 20 s starting at time 0 (pink area). Again, the two top panels represent V_t_ and RR, whereas the two bottom panels represent mABP and HR, respectively; responses are calculated as % change from baseline levels. (**D**) Schematic illustration of the stimulation/blocking paradigm to identify optimal KHFAC blocking parameters (top panel) with a corresponding and representative V_t_ trace (bottom panel). Stimulation at the respiratory response threshold was delivered for 60 s (pink bars), whereas KHFAC blocking at 20 kHz was applied in an incremental step-up fashion (dark grey columns) until the V_t_ stimulation response returned to baseline. Validation of the blocking parameters was then performed by applying a 30 s period of KHFAC block while stimulating at the respiratory response threshold for 20 s, starting 20 s after initiation of the block. When successful block of the electrical stimulation was confirmed, the identified blocking amplitude was used for blocking the pharmacologically evoked response. A 30 s period of KHFAC block was applied, and NaCN (pink arrow) was injected after 20 s. Confirmation of chemo-afferent nerve integrity was assessed by NaCN injection alone producing a characteristic increase in V_t_ post blocking.

Firstly, a protocol for pharmacological activation of the CB/CSN system using NaCN^10^ was established, evoking chemo-afferent CSN signal conduction (Fig. 2A,B). In all animals (n = 9), intra-carotid NaCN injections evoked a sharp increase in tidal volume (V_T_) peaking at 8–10 seconds (peak response lasting 4–5 s; mean peak response 162% of baseline; range 135.7–220.1%), accompanied by an inversely correlated decrease in respiratory rate (RR; mean peak response; 64.5% of baseline; range 48.3–81.9%). Cardiovascular responses were less pronounced, demonstrating a moderate increase in mean arterial blood pressure (mABP; mean peak response 119.6% of baseline; range 108.3–132.7%) peaking at 15–18 s with small changes in heart rate (HR; peak response 106.5% of baseline; range 102–111.7%). Intra-carotid injections of an identical volume of saline did not evoke cardiorespiratory changes (peak responses V_T_ 102.4% of baseline; RR 98.4%; mAPB 101.8% and HR 103.0%, respectively; data not shown).

### Electrical CSN stimulation at the respiratory response threshold mimics NaCN responses

**Figure 3:**
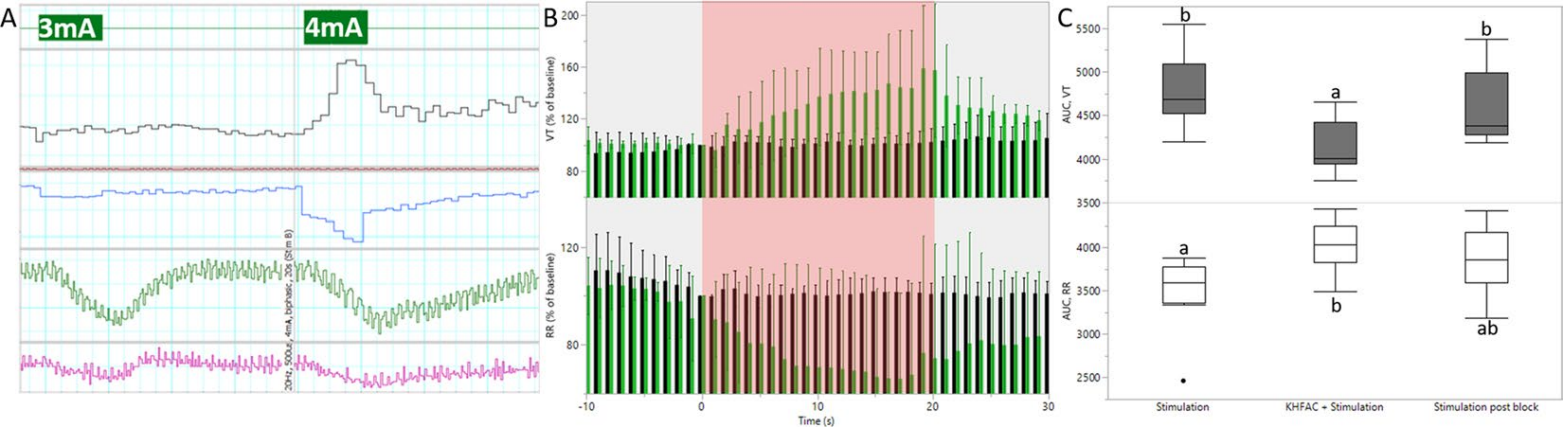
Electrically evoked stimulation responses. (**A**) Baroreflex activation evoking cardiovascular responses were observed at a lower stimulation current amplitude than the respiratory response threshold. In this animal, stimulation at 3 mA evoked a decrease in mABP (green trace; mm Hg), whereas no changes in V_t_ (black trace; l/min) or RR (blue trace; bmp) were seen. Stimulating at 4 mA evoked similar baro responses, but also produced an increase in V_t_ with a concurrent decrease in RR, and this stimulation amplitude was then identified as the respiratory response threshold. At both stimulation amplitudes, a small decrease in HR (pink trace; bpm) was seen. (**B**) Respiratory responses (group mean and 95% CI) when stimulating at the respiratory response threshold (pink area); green bars represent initial stimulation responses, whereas black bars represent responses while applying KHFAC block. Top panel: V_t_ (% change from baseline). Bottom panel: RR (% change from baseline). (**C**) Box plot illustrating AUC of the respiratory responses at the initial stimulation events (pre-blocking); at the blocking/stimulation paradigm; and at the stimulation events post block. Top panel: V_t_. Bottom panel: RR. Different letters denotes significant differences between interventions; V_t_ was significantly smaller during the blocking/stimulation paradigm than during the initial stimulation (*P* = 0.011) and when testing stimulation after the blocking/stimulation event (*P* = 0.034). RR was significantly higher during the blocking/stimulation paradigm than during the initial stimulation (*P* = 0.014).

Due to the short duration of the pharmacologically induced CB/CSN peak response, an electrical CSN stimulation paradigm to evoke a chemo-afferent response similar to NaCN was developed, that could be prolonged but quickly and easily reversed. Two bipolar cuff electrodes were placed on the CSN, and stimulation at 20 Hz was delivered through the distal of these (Fig. 2A). The stimulating current amplitude was titrated until respiratory changes mimicking the NaCN responses were seen. The resultant current amplitude, which for individual animals was in the range of 0.2–4 mA, was defined as the respiratory response threshold. When titrating stimulation current amplitudes to identify this threshold, baroreceptor activation evoking cardiovascular responses such as a decrease in mABP, was seen at lower current amplitudes than the respiratory responses (Fig. 3A). In all animals (n = 9), stimulation of the CSN at the respiratory response threshold evoked consistent respiratory responses for the duration of the stimulation period; mean peak V_T_ response was 160.7% of baseline (range 136.6–185.9%), accompanied by a decrease in RR (mean peak response 62.9% of baseline; range 33.0–80.5%; Fig. 3B). At the respiratory response threshold, only slight cardiovascular responses were seen (mean peak mABP response 100.7% of baseline; range 81.7–123.7%; and mean peak HR response 96.9% of baseline; range 88.5–108.7%; Fig. 3B).

### KHFAC successfully blocked the pharmacologically induced CB/CSN activation

Next, a model to select and test KHFAC blocking parameters was generated. Electrical stimulation was applied to elicit a prolonged chemo-afferent response (60 s) (Fig. 2D). After 10 s of evoked response, KHFAC (20 kHz) was applied through the proximal bipolar cuff electrode, at increasing amplitudes every 10 s, as detailed in methods section, and was removed for the last 10 s of the evoked response. This paradigm was used to identify a set of parameters able to cause a suppression or reduction in the electrically evoked respiratory response. When the optimised KHFAC amplitude was identified, conductance-block was confirmed by reversing the order of stimulation and block (Fig. 2D). When this block was successful (Fig. 3B,C), the same blocking parameters (12–35 mA) were used for blocking the pharmacologically evoked chemo-afferent response. Finally, NaCN delivered alone was used for confirming post-block CB/CSN integrity (Fig. 2D).

**Figure 4:**
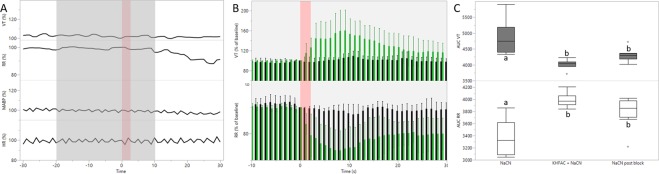
Chemo-afferent blocking. (**A**) Cardiorespiratory responses during successful KHFAC blocking (grey area) of the anticipated NaCN response (intra-carotid NaCN injection - pink area); the two top panels represent V_t_ and RR, whereas the two bottom panels represent mABP and HR, respectively; responses are calculated as % change from baseline levels. (**B**) Respiratory responses (group mean and 95% CI) to intracarotid NaCN injection (pink area); green bars represent initial chemo-afferent responses (pre-blocking), whereas black bars represent responses while applying KHFAC block. Top panel: V_t_ (% change from baseline). Bottom panel: RR (% change from baseline). (**C**) Box plot illustrating AUC of the respiratory responses for the initial NaCN injections (pre-blocking); when applying the blocking/NaCN paradigm; and when testing NaCN injections post block. Top panel: V_t_, bottom panel: RR. Different letters denotes significant differences (*P* < 0.05) between interventions.

Using this paradigm, complete block of the pharmacologically evoked response was observed in 3/9 animals (mean peak V_t_ response was 104.9% of baseline levels; whereas mean peak RR response was 92% of baseline, respectively; Fig. 4A). Partial block was seen in 3 animals (mean peak V_t_ response at 131.7% of baseline levels, and mean peak RR response at 85.5% of baseline levels), whereas the paradigm of blocking the pharmacologically evoked response could not be tested in the remaining 3 animals as stimulation responses were lost prior to obtaining stimulation block. In these animals, the pharmacologically induced response was also lost on the operated side, whereas contralateral intra-carotid NaCN injection evoked cardiorespiratory responses (data not shown), demonstrating the integrity of the un-manipulated naïve CB/CSN system.

Responses of all 6 animals in which blocking the pharmacologically evoked response could be tested are illustrated in Fig. 4B. The AUC for the NaCN induced V_t_ response was significantly smaller during blocking than during the pre-block NaCN injection (Fig. 4C**;** *P* = 0.001) whereas the AUC for RR was significantly larger during block than during the pre-block injection (*P* = 0.001). When re-testing the pharmacological CB/CSN response after successful block, respiratory responses to NaCN returned to pre-block levels in 2/6 animals (mean peak V_t_ responses 151.6% of baseline, mean peak RR responses 68.5% of baseline, respectively). In the remaining 4 animals, NaCN responses were diminished post-block, but still evident (mean peak V_t_ responses 113.2% of baseline, and mean peak RR responses 92.6% of baseline, respectively). As a group, AUC of the NaCN induced V_t_ response was significantly smaller post-block versus pre-block (*P* = 0.018) with a mean peak response post-block of 126% of baseline (range 104.1–140.6; Fig. 4C). Conversely, AUC for the pharmacologically evoked RR response was significantly higher pre-block than post-block (*P* = 0.008) with mean peak response post-block of 84.6% of baseline (range 67.1–97.6%).

## Discussion

To the best of our knowledge, this is the first report applying KHFAC block of the CSN in an anatomically- and physiologically-relevant translational model. Results from the current study demonstrate that pharmacological and electrical activation of a chemo-afferent CSN response is reproducible in anesthetised pigs. More importantly, blocking such activation through KHFAC modulation is feasible in acutely anesthetised pigs, with some limitations due to variability in the blocking efficiency. These observations may have important clinical implications and should warrant further model refinement prior to clinical use.

Using respiratory responses as a real-time biological marker for blocking effect was essential for the current model development, enabling titration of the appropriate KHFAC amplitudes and thresholds. Intra-carotid injection of NaCN evoked repeatable respiratory responses characterised by an increase in V_t_ with a concurrent decrease in RR. However, the short duration of the peak response limited its usefulness for testing and refining an optimal blocking paradigm, as this process entailed ramping through increments in high frequency current amplitudes for identifying the optimal blocking parameters. Therefore, we developed a paradigm of mimicking the chemo-afferent pharmacological responses by electrical stimulation, where peak respiratory responses were maintained for the duration of the stimulation period. Electrical stimulation was used for identifying the optimal blocking current amplitudes, which were subsequently used in the next part of the experiment. During this process, however, stimulation effects were lost in a third of the animals. In these animals, contralateral NaCN injection demonstrated the integrity of the un-manipulated CB/CSN system. Lack of response on the operated side should therefore be considered a procedural complication, where either surgical manipulation (from nerve isolation and cuff implantations) or current injection may have caused neural damage. On the other hand, blocking the pharmacologically evoked response was successful and repeatable in the subset of animals where this paradigm could be tested. However, complete block was only obtained in half of the animals, whereas a suppressed, but still detectable response was observed in the other half. A similar observation was made by Patel *et al*.^4^, where incomplete vagal nerve block was observed in a subset of animals in a rodent model of vagal nerve stimulation and block. No distinguishing features or event were observed suggesting why blocking failed in these animals^4^.

Post-blocking pharmacological chemo-afferent responses reached pre-blocking levels in 2/6 animals only, indicating that in the majority of animals, blocking resulted in a carry-over effect, the duration of which is unknown. In frog sciatic nerve, post-block recovery occurred in two distinct phases with complete block observed in the first phase, followed by gradual or abrupt block reversal in the second phase^11^. In addition to blocking parameters such as frequency^12^, amplitude^11,12^ and duration^11^, recovery dynamics from KHFAC block likely also depend on the nature of the target nerve. In rats, functional block of the CB/CSN system was observed 20 minutes after cessation of the KHFAC modulation whereas normal hypoxic responses were restored at 10 days post-blocking interruption^3^. Beyond these observations, it is currently unknown at which point in time after applying KHFAC block normal CB/CSN responsiveness is expected to return, and further studies are required to elucidate this.

From a biological perspective, the observed variability in blocking the chemo-afferent response may be explained in part by the anatomical diversity of the porcine CSN, corresponding to the diversity described in humans^6^. In fact, in humans suffering from carotid sinus syndrome, high CSN resection is not recommended precisely due to the anatomical complexity as multiple branches may be missed and consequently be left intact, impacting surgical outcome^6^. Although care was taken to include all identified CSN branches in the implanted cuff electrodes in the current experiment, smaller branches and interconnections to other nerves may have gone unnoticed and could account for a partial blocking response. Additionally, due to the multi-fascicular nature of the porcine CSN, with increased amounts of connective and adipose tissue compared to the rat, the uniformity of the conductances in the structure will change, such that some axons could be stimulated whereas others are blocked for the same charge induced, resulting in variable responses.

The macroscopic complexity of the porcine CSN is also reflected at the micro-anatomical and neurochemical level. The CSN consist of two types of baro-afferents; larger myelinated A-fibres mediate dynamic control of blood pressure and smaller A – and unmyelinated C-fibres relay tonic pressure control^13^. However, the majority of CSN fibres consist of small, unmyelinated chemo-afferent axons^5,8,14^, which amongst a plethora of neurotransmitters involved in CSN signal conduction, demonstrates immuno-reactivity towards TH and CGRP^15^. Additionally, acetylcholine plays a major role in generating the CB chemo-afferent discharge^16^. In the current study, co-localisation of ChAT + and TH + axons were present within some fascicles, whereas other fascicles had a distinct separation between these two. Small numbers of CGRP + axons were irregularly distributed throughout all investigated fascicles, with small amounts of clustering. There was no evidence of spatial grouping of chemo-afferents within the nerve. Due to this mixed nature of the nerve, and as demonstrated by others, electrical stimulation of the CSN concomitantly activates baro- and chemo-afferents^17^. In the current study, we observed baro-afferent activation at a lower stimulation current amplitude than the respiratory response threshold, reflecting the larger size or myelinated status of the dynamic baro-afferent fibres compared to the chemo-afferents. Baro-reflex activation inhibits the sympathetic activity and increases the parasympathetic drive to the heart, ultimately resulting in reduced mABP^18^. In normotensive subjects, altering blood pressure as a consequence of CSN modulation would represent an off-target adverse effect. However, when stimulating at the respiratory response threshold, cardiovascular parameters remained stable. This observation can be explained by the fact that chemoreceptor stimulation not only evokes respiratory effects, but also increases sympathetic activity, counteracting the baro-reflex.

Selective baro-reflex activation through electrical stimulation of the carotid sinus, has been investigated for treating drug-resistant hypertension^18,19^. Although commercially available, several concerns regarding safety and efficacy of this treatment modality have been raised^18,20^. Although none of these were related to the stimulation parameters (i.e. charge density or injected current) but rather were procedural or device related, the situation may be different for a treatment based on electrical block due to higher energy requirements, as investigated in the current study. Considering our results, we believe that further investigation of reliability of CSN KHFAC block is warranted.

From an engineering perspective, hardware issues such as cuff-fit and electrode design are likely to contribute to the observed variability in blocking response. The large range of nerve length and diameter led to variability in cuff fit and therefore also circumferential electrode coverage between animals. Optimal cuff design should try to limit current leakage, a non-uniform electric field, improve electrode-axon integration, and circumferential fit, that may have occurred in these experiments and contributed to variability and lack of reversibility and reproducibility seen in the animals where stimulation effects were lost.

In conclusion, reversible neural block using KHFAC of the porcine CSN is feasible and successful from a procedural and engineering perspective. This has enabled us to translate this technology from rodent experiments into the realm of an anatomically relevant species for the clinic; enabling safety studies and cuff design to be directed toward patient treatment. However, many refinements are needed along the way and additional large animal studies are warranted to determine the biological significance of blocking the CSN.

## Methods

All animal studies were ethically reviewed and carried out in accordance with Animals (Scientific Procedures) Act 1986 and the Galvani Policy on the Care, Welfare and Treatment of Animals. The protocol was approved by the Royal Veterinary College Animal Welfare and Ethical Review Board and the Galvani Animal and Scientific Review Committee.

### Anatomical study – gross anatomy and histology of the porcine CSN

Detailed cadaveric dissections of the left and right CSN region were performed in 6 farm pigs (Large white/British landrace cross, body weight 45–50 Kg). The number of CSN branches as well as length and diameter per branch were recorded. The CSN with all branches and the carotid tree bifurcation was harvested and placed in 10% neutral buffered formalin (NBF; VWR, UK) for histological analyses.

Routine staining with Haematoxylin and Eosin (H&E) was performed, and digital images of the H&E sections were acquired at 2x magnification and captured with appropriate software (Image J 1.50i). Every single nerve bundle comprising the CSN was manually selected by using the software ROI manager function and the number of bundles was recorded, and bundle size was assessed by measuring Minimum Feret’s diameter (µm).

For immunohistochemistry, 4 µm paraffin embedded sections were dewaxed prior to heat antigen retrieval in citrate buffer pH 6.0. In brief, sections were incubated with tyrosine hydroxylase antibody (Mouse monoclonal anti TH, Abcam ab129991; dilution 1:2000) and acetylcholine transferase antibody (Goat polyclonal anti ChAT, Millipore AB144; dilution 1:200); or TH and calcitonin gene-related peptide antibodies (Goat anti GCRP, Abcam AB36001; dilution 1:3000); or neurofilament 200 and myelin basic protein antibodies (Rabbit polyclonal anti NF200, Abcam ab8135; dilution 1:1000 and rat monoclonal anti MBP; Abcam ab7349; dilution 1:200). Fluorescent conjugated secondary antibodies were then incubated against the relevant host primary antibody. Alexafluor 488 and 594 nm secondary antibody (ThermoFisher) combinations were used to distinguish between the pairs of primary antibodies. Cell nuclei were counterstained with 4′,6-diamidino-2-phenylindole (DAPI). For each section, two different digital images were randomly captured at 20x magnification, and pseudocoloured composites generated using appropriate software (AxioVision LE64). The proportion of positive fibres were quantified by manual counting in an area of 100 × 100 µm.

For semi-thin resin sections, samples were fixed for a minimum of 48 hours in NBF before being processed. Thereafter, samples were washed in 0.1 M cacodylate buffer before a second fixation in 1% osmium tetroxide (Agar Scientific, Stansted UK) for 3 hours at 4 °C. After washing in 0.1 M cacodylate buffer, the samples were dehydrated through a graded series of ethanol with a final step in propylene oxide (PO). They were then infiltrated with mixtures of epoxy resin and PO before being embedded in neat resin. The blocks were then polymerised at 60 °C for 16 hours. Semi-thin sections (1 µm) were cut and mounted on glass slides before being stained with 1% toluidine blue (Thermo Scientific, UK) and cover-slipped with DPX. The proportion of myelinated versus unmyelinated axons was estimated from semi-thin sections and immunohistochemistry sections.

### Animals

All procedures for the animal experiments were in accordance with the animal protocols approved by the United Kingdom Home Office. Based on our data demonstrating similar gross and histological anatomy of the porcine CSN region to the human counterpart (Fig. 1), nine female farm pigs (Large white/British landrace cross, body weight 45–50 Kg; approximately 12 weeks old) were sourced from a commercial pig farm and acclimatised at the research facility for a minimum of 7 days prior to the experiment. Animals were group housed on straw bedding and given food and water ad libitum until 12 hours prior to the experiment, at which point food was withheld.

### Surgical procedures

The experimental set-up is detailed in Fig. 2A. On the day of the experiment, animals were pre-medicated with ketamine (20 mg/Kg) and midazolam (0.5 mg/Kg) administered by intramuscular injection. Fifteen minutes after premedication, a 20 G intravenous catheter was placed in the auricular vein, and general anaesthesia was induced by administering propofol (2 mg/Kg) intravenously. Animals were subsequently intubated with an endotracheal tube, allowing anaesthesia to be maintained with sevoflurane vaporised in a 50:50 mixture of oxygen and medical air. After induction of general anaesthesia, the animal was positioned in dorsal recumbency, and a left-sided indwelling jugular vein catheter as well as a right-sided femoral arterial catheter were both placed under ultrasonographic guidance. Animals were repositioned into left lateral recumbency and the right lateral cervical area was clipped and prepared for aseptic surgery. Surgical field infiltration of local anaesthesia (2% Lidocaine, 10–12 ml) was allowed to take effect for a minimum of 5 minutes prior to placing the skin incision. Volume-controlled mechanical ventilation was maintained for surgery and implantation, after which animals were switched to spontaneous ventilation. Ventilation was monitored using a spirometer measuring tidal volume (V_T_; ml) and respiratory rate (RR; breaths per minute), whereas circulation was monitored by electrocardiogram and invasive arterial blood pressure from which heart rate (HR, beats per minute) and mean arterial blood pressure (mABP; mm Hg) were calculated. Additional parameters were collected including end-tidal CO_2_, fraction of inspired oxygen, end-tidal sevoflurane, pulse oximetry and core body temperature. All parameters were digitally recorded using a 16 channel PowerLab acquisition system (AD Instruments) with LabChart 8 software at 2 kHz sampling frequency.

The right common carotid artery, carotid sinus and CSN were accessed through a 20 cm long J-shaped lateral incision placed 1 cm caudal to the vertical ramus of the mandible, extending in a caudal direction at the level of the ventral border of the trachea. The incision continued through all tissue planes until identification of the internal jugular vein and carotid sheath. An intra-arterial catheter (22 G, 25 mm catheter connected to an extension set with volume 0.3 ml) intended for NaCN injection was placed in the common carotid artery, and fixed in place using fibrin glue (Tisseel, Baxter Healthcare, Compton, Newbury, UK). Careful dissection cranial to the carotid bifurcation revealed the CSN branching off the glossopharyngeal nerve and travelling towards the carotid sinus. The number of identified CSN branches were recorded and subsequently dissected free of fatty and loose connective tissue prior to implantation of two bipolar cuff electrodes of appropriate size (CorTec AirRay Micro Cuff Tunnel; CorTec GmbH, Freiburg, Germany); when feasible, all identified branches were cuffed together. Fibrin glue was used to secure the cuffs and to prevent current spread from the cuff ends. The proximal (towards the brain) of these electrodes was intended for applying KHFAC block, whereas the distal (towards the CB) was intended for CSN stimulation. At the end of the experiment, animals were humanely euthanised with an overdose of pentobarbital administered intravenously.

### Pharmacological CB/CSN activation and KHFAC blocking

An optimal dose of intra-carotid administration of NaCN evoking chemo-afferent activation of the CSN, and within the dose range previously reported in this species^10^ was identified in preliminary experiments. This dose (0.04 mg/kg, total volume 1.00 ml, followed by 1 ml saline flush, administered into the carotid catheter; injection time < 2 s) consistently and repeatedly evoked cardiorespiratory responses (Fig. 2B) characterised by an increase in tidal volume (Vt) with a corresponding decrease in respiratory rate (RR), and was subsequently used for the experiment. A minimum of two NaCN injections, alternated with administration of saline (identical volume and injection speed) were performed prior to developing a paradigm of electrical activation of the chemo-reflex. Electrical stimulation of the CSN was achieved by applying symmetric biphasic pulses at pulse width 1000 µs (Digitimer DS5 Isolated Bipolar Current Stimulator, Digitimer Ltd, Welwyn Garden City, UK) through the distal cuff electrode at 20 Hz for 20 s; optimal stimulation frequency was identified in preliminary experiments. Cardiorespiratory responses were allowed to return to baseline levels between each 20 s stimulation period. Individual current amplitude titration was performed until repeatable respiratory effects mimicking the chemo-afferent responses evoked by NaCN were observed (Fig. 2C). The resultant current amplitude was then defined as the respiratory response threshold, which was subsequently used for identifying and optimising KHFAC blocking parameters.

KHFAC block was delivered by a high precision floating AC current source (Keithley 6221, Tektronix, Beaverton, OR) with a low noise triax cable (Keithley 237-ALG-2, Tektronix). To filter DC contamination of AC delivery, a circuit containing DC-blocking capacitors and DC-shunting inductors was placed in parallel with the electrode on the output of the current source^21^. The reported current amplitudes are peak-to-peak. In brief, the stimulation/blocking paradigm consisted of stimulating the CSN through the distal cuff electrode for 60 s at the respiratory response threshold. Optimal blocking frequency was identified in preliminary experiments, and KHFAC blocking at 20 kHz was applied through the proximal electrode 10 s after initiating the stimulation. Blocking was delivered in an incremental step-up fashion starting at 3 mA, increasing the current amplitude in 1 mA steps (3 times) every 10 s, until the expected stimulation response was visibly diminished or no longer evident (Fig. 2D). Again, cardiorespiratory responses were allowed to return to baseline levels between each 60 s stimulation/blocking event.

When the optimal parameters for KHFAC blocking were identified as described above, these parameters were validated for blocking the electrical stimulation prior to developing a paradigm for blocking the chemo-afferent NaCN response. In brief, a 30 s period of KHFAC blocking was applied with the stimulus (20 s of electrical stimulation) delivered 20 s after initiation of the block. Again, cardiorespiratory responses were allowed to return to baseline levels between each intervention, and electrical stimulation was delivered between each blocking/stimulation event to test nerve integrity. When successful block of the electrical respiratory response threshold was confirmed, blocking the chemo-afferent NaCN response was attempted. For this paradigm, a 30 s period of KHFAC blocking was applied while NaCN was injected 20 s after initiation of the block (Fig. 2D). To test nerve integrity, NaCN was administered between every blocking/chemo-afferent stimulation event, and this sequence (KHFAC + NaCN; then NaCN alone) was repeated at least twice when chemo-afferent responses were still evident.

When chemo-afferent responses were lost, additional attempts at evoking responses with either pharmacological or electrical stimulations were made up to 270 minutes after losing the response. If the response was still no longer present, a contralateral intra-carotid NaCN injection was performed to test the integrity of the naïve CB/CSN system.

### Statistical analyses

Respiratory (V_T_ and RR) and cardiovascular (mABP, HR) responses were calculated as a % of baseline; baseline was defined as the value at time 0 when delivering the stimulus. For each test condition, a group mean and 95% confidence interval was constructed; also, mean peak responses were recorded (maximum responses for V_T_, mABP and HR; minimum responses for RR). For statistical purposes, the area under the curve (AUC; standardised to the timeframe from 10 s prior to administering the stimulus to 30 s after delivering the stimulus) was calculated for the cardiorespiratory responses (V_T_, RR, mABP, HR) and for all interventions (NaCN; saline; electrical stimulation; stimulation + KHFAC blocking; KHFAC blocking + NaCN; NaCN post KHFAC blocking). Response differences between interventions were analysed using a mixed model with Tukey HSD all pairwise comparisons, with intervention as fixed effect, and animal as random effect. Statistical significance was defined as *P* < 0.05.

## Data Availability

The datasets generated and analysed during the current study are available from the corresponding author on reasonable request.
